# NHERF1 regulates the progression of colorectal cancer through the interplay with VEGFR2 pathway

**DOI:** 10.18632/oncotarget.13949

**Published:** 2016-12-15

**Authors:** Yanan Gu, Hefen Yu, Chengcheng Hao, Tracey A Martin, Rachel Hargest, Junqi He, Shan Cheng, Wen G Jiang

**Affiliations:** ^1^ Department of Biochemistry and Molecular Biology, Capital Medical University, Beijing 100069, China; ^2^ Beijing Key Laboratory of Cancer & Metastasis Research, Capital Medical University, Beijing 100069, China; ^3^ Cardiff China Medical Research Collaborative, Cardiff University School of Medicine, Heath Park, Cardiff CF14 4XN, UK

**Keywords:** colorectal cancer, NHERF1, VEGFR, hypoxia, PDZ protein

## Abstract

The oncogenic role of ectopic expression of Na^+^/H^+^ exchanger regulatory factor 1 (NHERF1) was recently suggested in colorectal cancer, where it was implicated in playing a role in the tumor hypoxia microenvironment. Here we showed that a high level expression of NHERF1 was found in colorectal cancer tissues and that the expression of NHERF1 was positively correlated with VEGFR2 expression. The prognostic value of VEGFR2 expression in colorectal cancer relied on the expression of NHERF1. The up-regulation of NHERF1 induced by the exposure to hypoxia in colon cancer cells depended on the activation of VEGFR2 signaling. NHERF1 in turn inhibited the activation of VEGFR2 signaling which could be regulated by the interaction between NHERF1 and VEGFR2, resulting in the reduction of migration and invasion of colon cancer cells. These results suggest a dynamic interplay between NHERF1 and VEGFR2 signaling in colorectal cancer, which could explain the contribution of NHERF1 to the regulation of tumor cell responses to the hypoxia microenvironment.

## INTRODUCTION

Despite very important therapeutic advances during the last decade, colorectal cancer (CRC) is the third most commonly diagnosed cancer in the world [1]. In 2016, new cases and deaths from CRC were estimated 134,490 and 49,190 in the United States, respectively [2]. Surgical resection of localized tumors improves patients’ survival [3], but over half of them will develop a recurrence [4]. There is still a real need for therapies that would reduce the risk of recurrence after surgery and chemotherapy treatment and prolong patient survival with metastatic disease.

In solid malignancies, fast proliferation can outgrow the supply of nutrients and oxygen provided by the malformed tumor vasculature [5]. Hypoxia is common in human cancers and grants stabilization of hypoxia-inducible factor 1α (HIF1α) [5, 6]. This transcription factor ensures cell survival through adaptive changes in cell metabolism [5]. Among several hypoxia related genes, the vascular endothelial growth factors (VEGFs) mediate their effects on proliferation and survival mostly through the VEGF receptor 1 (VEGFR1) and 2 (VEGFR2) within endothelial cells [7, 8]. Nevertheless, it has been demonstrated that VEGFR1 and VEGFR2 expression in CRC cells correlated significantly with high risk of metastasis and relapse [9, 10].

VEGFs and their receptors are targets for cancer drug therapy [11]. Blockade of the VEGF/VEGFR pathway has shown efficacy and led to improvements in survival in CRC [12, 13]. However, patient responses to anti-VEGF/VEGFR therapies are variable within and across indications. Most of the reported outcomes are averages but in reality these vary quite extensively from complete response to no response [14, 15]. Many patients progress after initial disease stabilization due to acquired resistance to anti-VEGF/VEGFR drugs [16, 17]. Thus, it is very important to elucidate the regulation of VEGF/VEGFR2 pathway *in vivo* and it is crucial to find predictive biomarkers to identify patients that may benefit from anti-VEGF/VEGFR therapies.

Na^+^/H^+^ exchanger regulatory factor 1 (NHERF1), also named ezrin radixin-moesin (ERM) binding phosphoprotein 50 (EBP50), is a scaffolding protein, contains two N-terminal tandem domains PDZ1 and PDZ2 and a C-terminal ERM domain [18]. NHERF1 can bind more than 30 proteins through PDZ domains [19], such as platelet-derived growth factor receptor (PDGFR) [20, 21], epidermal growth factor receptor (EGFR) [22], β-catenin [23]. Recently, it has been reported that NHERF1 is involved in cancer progression, including breast cancer [24], hepatocellular carcinoma [23] and glioblastoma [25]. Other studies have showed that NHERF1 is considered a new player in colorectal cancer progression [26]. It has been reported that nuclear NHERF1 expression probably contributes to the malignant phenotype during the early stages of carcinogenesis [27, 28]. Also, cytoplasmic NHERF1 was higher in primary cancer than in adjacent normal mucosa, and tumors overexpressing NHERF1 were associated with nodal and distant metastases, poor grade and lymphovascular invasion (LVI) [29]. It was also reported that expression of NHERF1 was strongly correlated with expression of HIF1α and VEGFRs both in breast cancer and lymphatic metastastic colorectal cancer [30, 31], indicating that NHERF1 expression might be involved in metastatic progression by regulating the cell adaptive changes to the tumor microenvironment. However, the mechanism of NHERF1 up-regulation in cancers and the contribution of NHERF1 to the regulation in the metastatic progression were still unclear.

The experiments from this study revealed that there could be negative feedback in colon cancer cells where NHERF1 up-regulation was induced by hypoxia, depending on the activation of VEGFR2 signaling pathway, in turn reducing the phosphorylation promoted activation of VEGFR2 signaling, resulting in the inhibition of migration and invasion of colon cancer cells. These results suggest that NHERF1 could regulate the progression of CRC through the interplay with VEGFR2 signaling pathway.

## RESULTS

### NHERF1 expression was associated with clinical status of colorectal cancer

**Figure 1:**
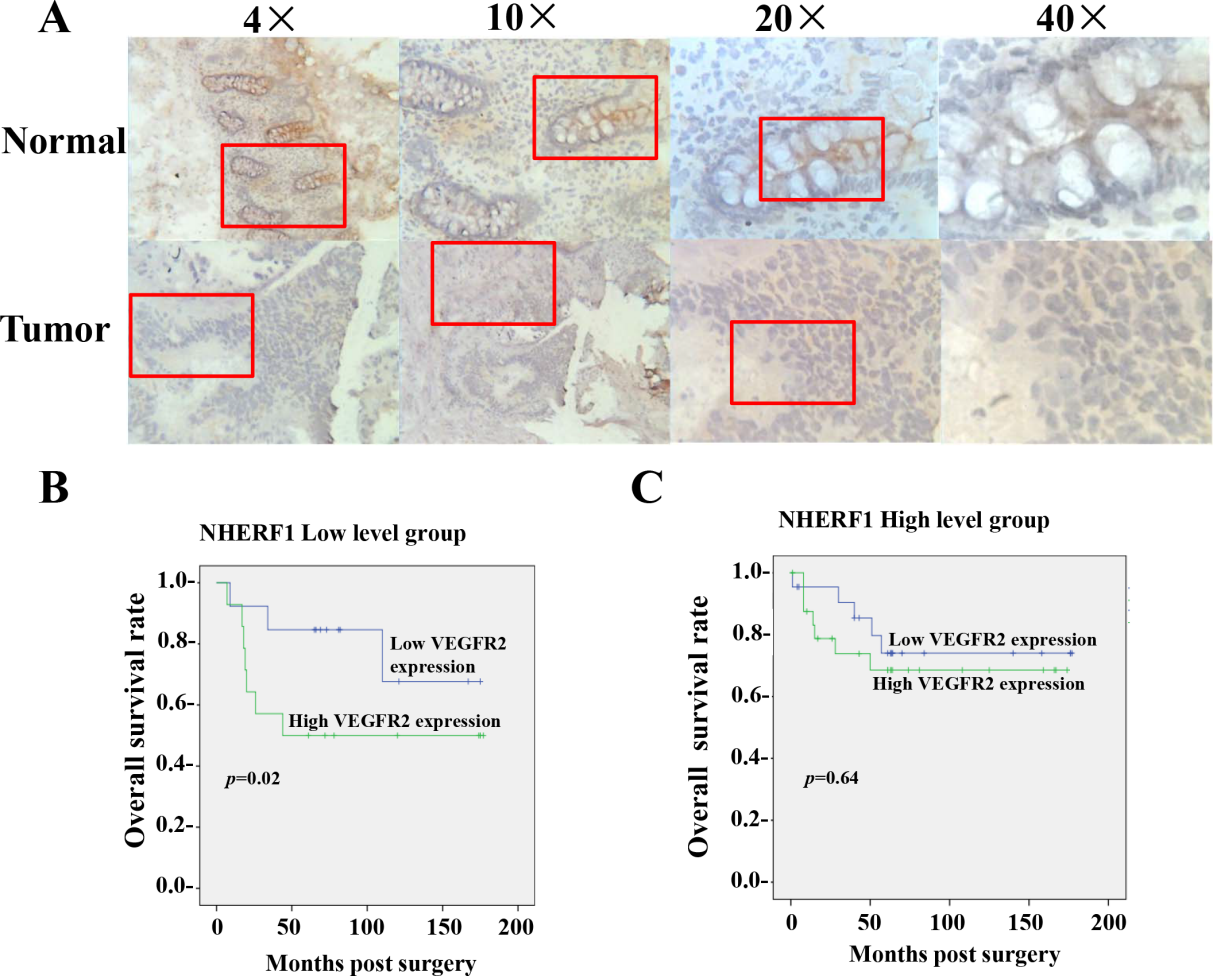
Prognosis value of VEGFR2 expression in colorectal cancer relied on the expression of NHERF1 The NHERF1 protein was readily detected in both normal and cancerous epithelial cells of colorectal tissues, but not in surrounding stromal cells by IHC (**A**). High expression of VEGFR2 was associated with shorter overall survival of patients with colorectal cancer of low NHERF1 expression (**B**). No significant differences were observed in the OS curve analysis between patients with high and low VEGFR2 expression in the patients with colorectal cancer of high NHERF1 expression (**C)**.

NHERF1 protein expression was readily detected in normal and cancerous epithelial cells of colorectal tissues, but not in surrounding stromal cells (Figure 1A). NHERF1 transcripts were quantified in colorectal tissues including 64 colorectal cancer tissues and 50 adjacent normal tissues. NHERF1 transcripts demonstrated a higher level in tumors (*p* = 0.0083, Tumor *vs* Normal) (Table 1). An elevated level of NHERF1 expression was detected in samples from patients with recurrence (*p* = 0.014, recurrence *vs* disease free). NHERF1 transcript levels were also increased in samples from patients with metastasis compared with those from disease free patients, though there was no significant difference (*p* = 0.079) (Table 1). However, no relationship was observed in the colorectal cancer tissues between NHERF1 expression and other clinic variables including Dukes and TNM stages (Table 1).

**Table 1:** Quantitative PCR analysis of NHERF1 expression in human colorectal tissues

Clinical data	Grouping	No.	Median	CI 95%	*p*-value	Mean ± SEM	*p*-value
Tissue sample	Normal	50	0.770			2.38 ± 0.56	
	Tumor	64	1.655	(−1.635,−0.121)	0.0083*	29 ± 15	0.093
Dukes stages	A	6	16.15			16.3 ± 6.1	
	B	29	1.14	(−0.36,25.33)	0.11	26 ± 21	0.67
	C	30	1.14	(−0.73,25.18)	0.10	30 ± 26	0.60
TNM stages	1	8	5.78			12.3 ± 5.2	
	2	27	1.25	(−1.28,20.85)	0.52	28 ± 23	0.51
	3	24	1.12	(−1.51,21.01)	0.62	38 ± 32	0.45
Node	Negative	35	1.37			24 ± 17	
	Positive	29	1.14	(−0.83,1.28)	0.98	31 ± 27	0.82
Survival status	Disease free	19	0.89			2.94 ± 1.1	
	Recurrence	19	3.96	(−12.89,−0.25)	0.014*	14.9 ± 5.6	0.051
	Metastasis	17	4.17	(−5.70,0.07)	0.079	8.7 ± 2.9	0.077
	Death	10	1.0	(−3.0,1.1)	0.89	63 ± 61	0.35
Differentiation	1	2	0.715			0.715 ± 0.66	
	2	48	1.410	(−25.529,1.254)	0.39	18.5 ± 13	0.17
	3	14	1.2	(−776.6,1.3)	0.47	62 ± 55	0.28

### Prognostic value of VEGFR2 expression in colorectal cancer relied on the expression of NHERF1

In previous work from our team, a high VEGFR2 level was found in colorectal cancer tissues compared to normal background tissues and was associated with disease progression [32]. In the present study, NHERF1 transcripts were quantified in the same tissue bank. A high level expression of NHERF1 was found in the colorectal cancer tissues and it was shown that NHERF1 expression was positively correlated with VEGFR2 expression (Table 2).

**Table 2:** The association of the expression of NHERF1 and VEGFs/VEGFRs in human colorectal tissues

	r vs NHERF1*	*p* value*
VEGF A	−0.10	0.36
VEGF B	0.01	0.87
VEGF C	0.005	0.96
VEGF D	−0.07	0.42
VEGFR1	0.10	0.26
VEGFR2	0.25*	0.004*
VEGFR3	0.02	0.83

* Correlation coefficients by Spearman ranked correlation test, *n* = 114.

The colorectal cancer tissues were separated to two subgroups, NHERF1 low level group and NHERF1 high level group, by the median value (2.0 copies) of NHERF1 transcripts quantified in colorectal tissues. The overall survival rates were analyzed in the two subgroups respectively. The mean OS was 99.3 [(58.4–140.2, 95% confidence interval (CI)] months in patients with high VEGFR2 expression levels (cut-off by median value (50 copies) of VEGFR2 transcripts) and 140.4 (106.6–174.1, 95% CI) months in patients with low VEGFR2 expression levels in the NHERF1 low level group (*p* = 0.02) (Figure 1B). However no significant differences were observed in the OS curve analyse (*p* = 0.64) between patients with high and low VEGFR2 expression in the NHERF1 high level group (Figure 1C).

### Exposure to hypoxia increased NHERF1 expression depending on the activation of VEGFR2 signaling pathway

**Figure 2:**
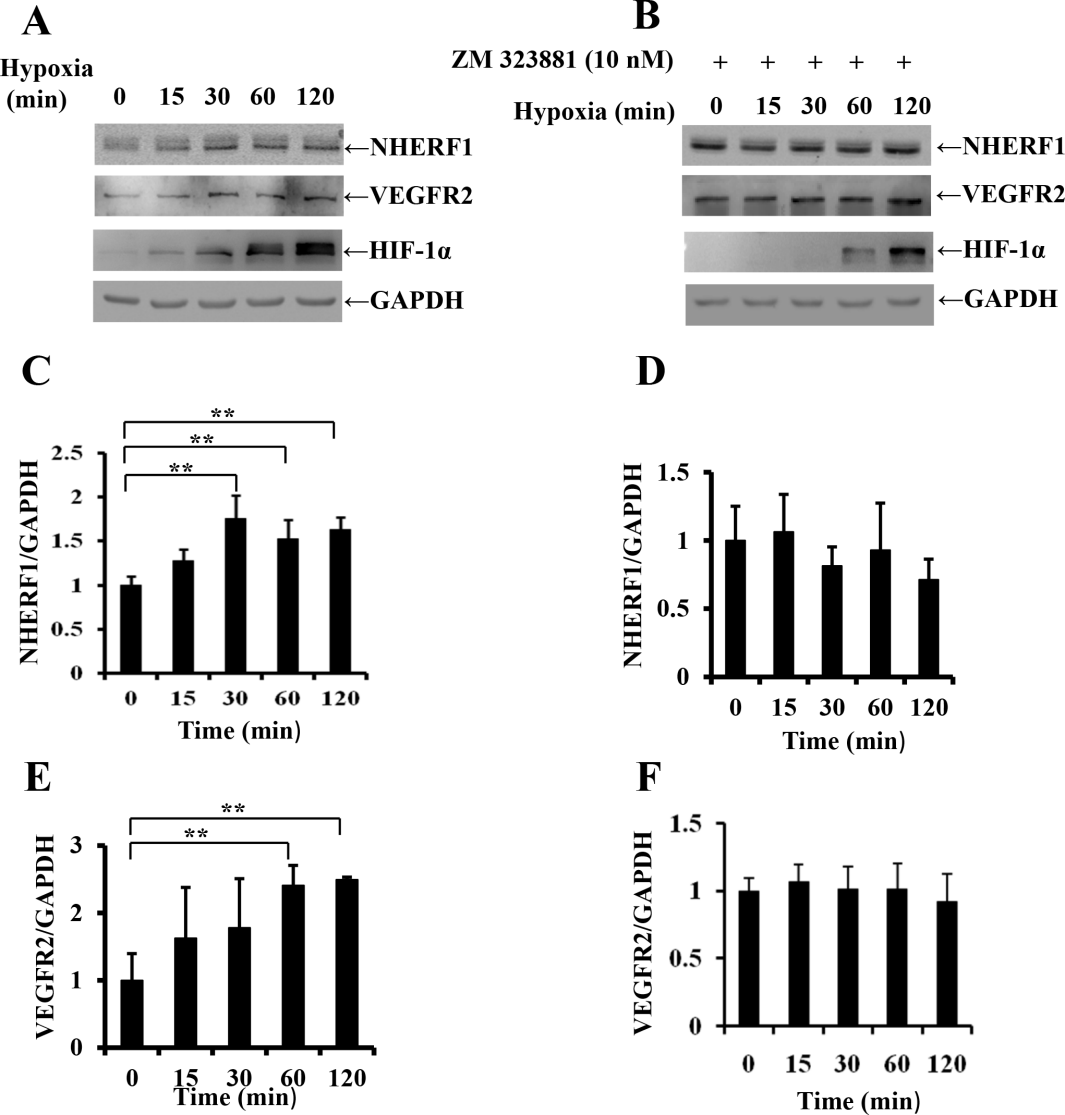
Exposure to hypoxia increased NHERF1 expression depending on the activation of VEGFR2 signaling pathway Confluent cell monolayer of the human RKO cells were exposed to hypoxia (1% O_2_) for 15, 30, 60, 120 min and treated with (**A**) or without (**B**) ZM323881 (10 nM), VEGFR2 special inhibitor. NHERF1 and VEGFR2 expression was compared with non-treated monolayers by Western analysis. Histograms underneath each blot represent the trend of NHERF1 or VEGFR2 expression with (**C**, **E**) or without (**D**, **F**) ZM323881 (10 nM) normalized to GAPDH for three independent experiments. **p < 0.01

We investigated the possible interaction between the hypoxia microenvironment and NHERF1 by subjecting colon cancer cells RKO to hypoxia. Western blot analysis revealed that exposure to hypoxia (1% O_2_) (Figure 2A) increased NHERF1 and VEGFR2 expression in RKO cells by the strong correlation with HIF-1α. In contrast, VEGFR2 special inhibitor ZM-323881 removed the up-regulation of NHERF1 and VEGFR2 induced by hypoxia (Figure 2B).

### Effects of NHERF1 expression on the migration and invasion of RKO cells

**Figure 3:**
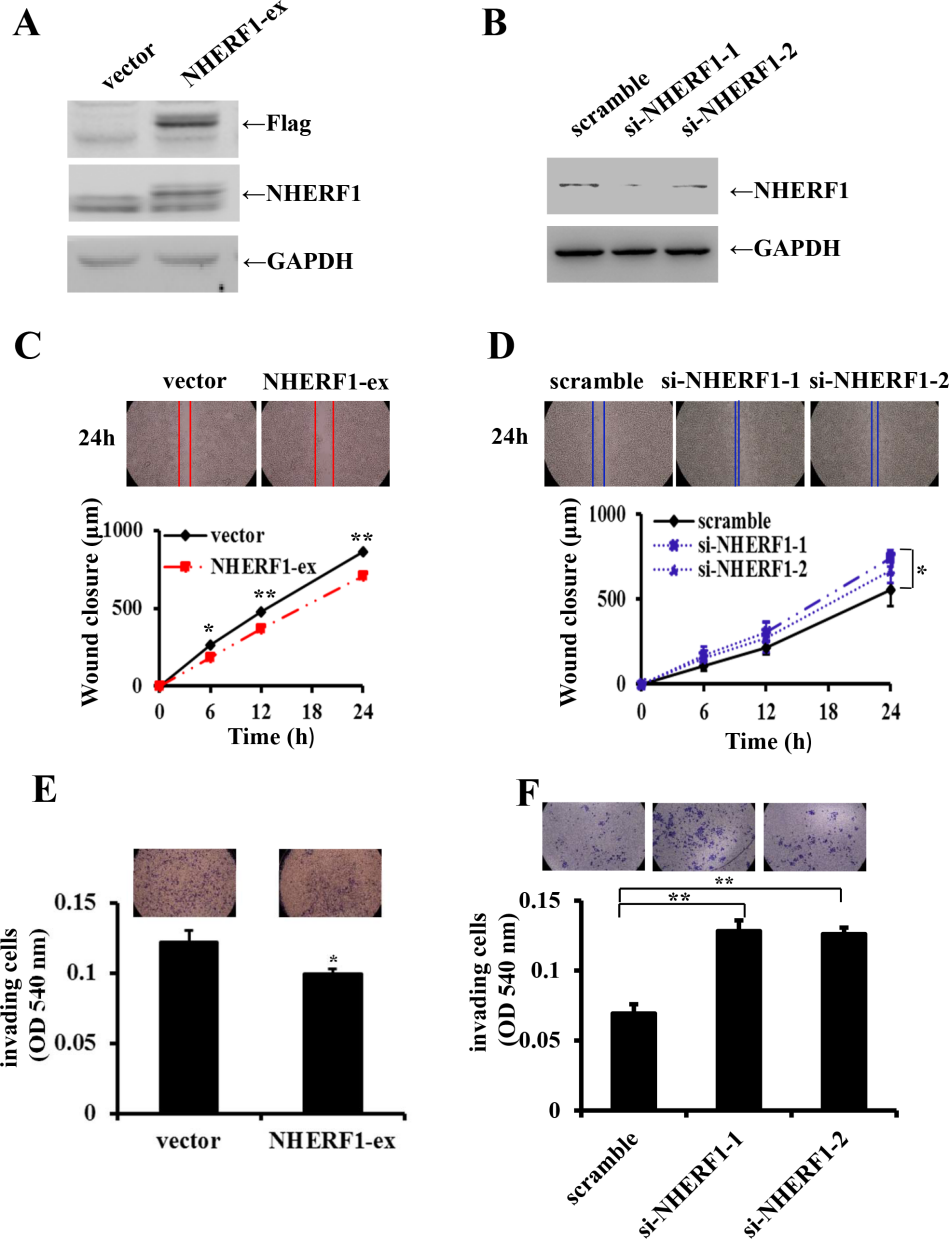
Effects of NHERF1 expression on the migration and invasion of RKO cells NHERF1 expression was detected in RKO cells transfected with flag-NHERF1 plasmid (**A**) and anti-NHERF1 siRNAs (**B**) The effects of NHERF1 overexpression and knockdown on cell migration (**C**, **D**) and invasion (**E**, **F**) were detected. The results represent the mean values ± SD of three independent experiments (C, D, E, F). *p < 0.05, **p < 0.01

The expression level of NHERF1 was considerably increased in Flag-NHERF1 plasmid transfected cells, compared with vector control cells (Figure 3A). Cells transfected with the anti-NHERF1 siRNAs exhibited a markedly reduced level of NHERF1 expression compared with the scramble control (Figure 3B). Compared with control cells, cell migration was inhibited by up to 75% in cells overexpressing NHERF1 at 24 h (Figure 3C). This was consistent with the observations in NHERF1 knockdown RKO cells, in which cells migration was increased up to 120% and 135% respectively (Figure 3D). Cell invasion was also reduced significantly in the NHERF1 overexpression cells (Figure 3E); Knockdown of NHERF1 expression resulted in a remarkable increase in invasive ability of RKO cells (Figure 3F). The inhibitory effect of NHERF1 on cell migration was also observed in colon cancer cells HRT-18 (Supplementary Figure S1A, S1B).

### NHERF1 interacted with VEGFR2 in cells

**Figure 4:**
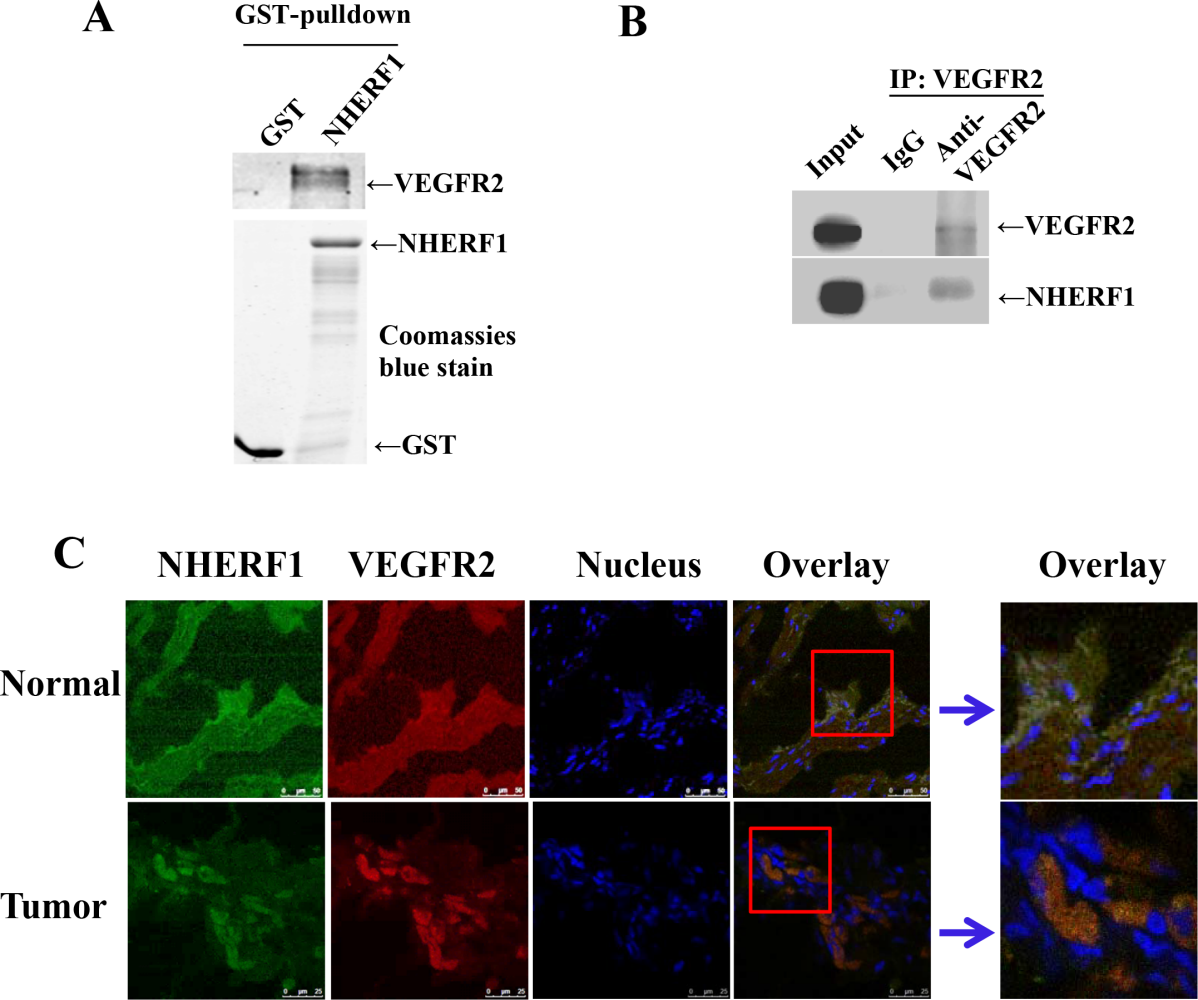
NHERF1 interacted with VEGFR2 Glutathione S-transferases (GST)–NHERF1 beads were incubated with RKO cell lysates, and then the pull-down complexes were subjected to VEGFR2 immunoblotting (**A**) Coomassie blue-stained gel was used to visualize the same amount of input of fusion proteins. NHERF1 and VEGFR2 interacted in cells (**B**). RKO cell lysates were subjected to immunoprecipitation with the anti-VEGFR2 antibody or IgG as the negative control. The lysates were probed with the anti-NHERF1 antibody or anti-VEGFR2 antibody to visualize the presence of NHERF1 and VEGFR2, respectively. NHERF1 co-localized with VEGFR2 in native tissues (**C**). Normal and cancerous human colorectal tissues were fixed, permeabilized and probed with anti NHERF1 (shown in green) and anti-VEGFR2 (red). Nuclei were stained with Hoechst 33258. Co-localization was analyzed by confocal microscopy. The data shown in all panels of this figure are representative of three independent experiments.

It was recently reported that VEGF_165_ bound to VEGFR2 and NP1 results in the formation of a complex, which is dependent on the bridge of Synectin, a PDZ domain-containing protein [33, 34]. As a PDZ protein, it seemed a reasonable possibility that NHERF1 might form a protein complex with VEGFR2 in cells. To test this hypothesis, we employed a fusion protein pull-down assay. As shown in Figure 4A, GST-NHERF1 was found to robustly pull down VEGFR2. VEGFR-2 immunoprecipitation from RKO cells followed by Western blot analysis revealed the formation of VEGFR2-NHERF1 complex in cells (Figure 4B). Immunofluorescence was used to examine the subcellular localization of NHERF1 and VEGFR2 in colorectal tissues. Representative images are shown in Figure 4C. VEGFR2 appeared to locate both in cytoplasm and membrane and being co-localized with NHERF1 (Figure 4C).

### NHERF1 inhibited VEGF_165_-induced VEGFR2, AKT and PLCγ activation

**Figure 5:**
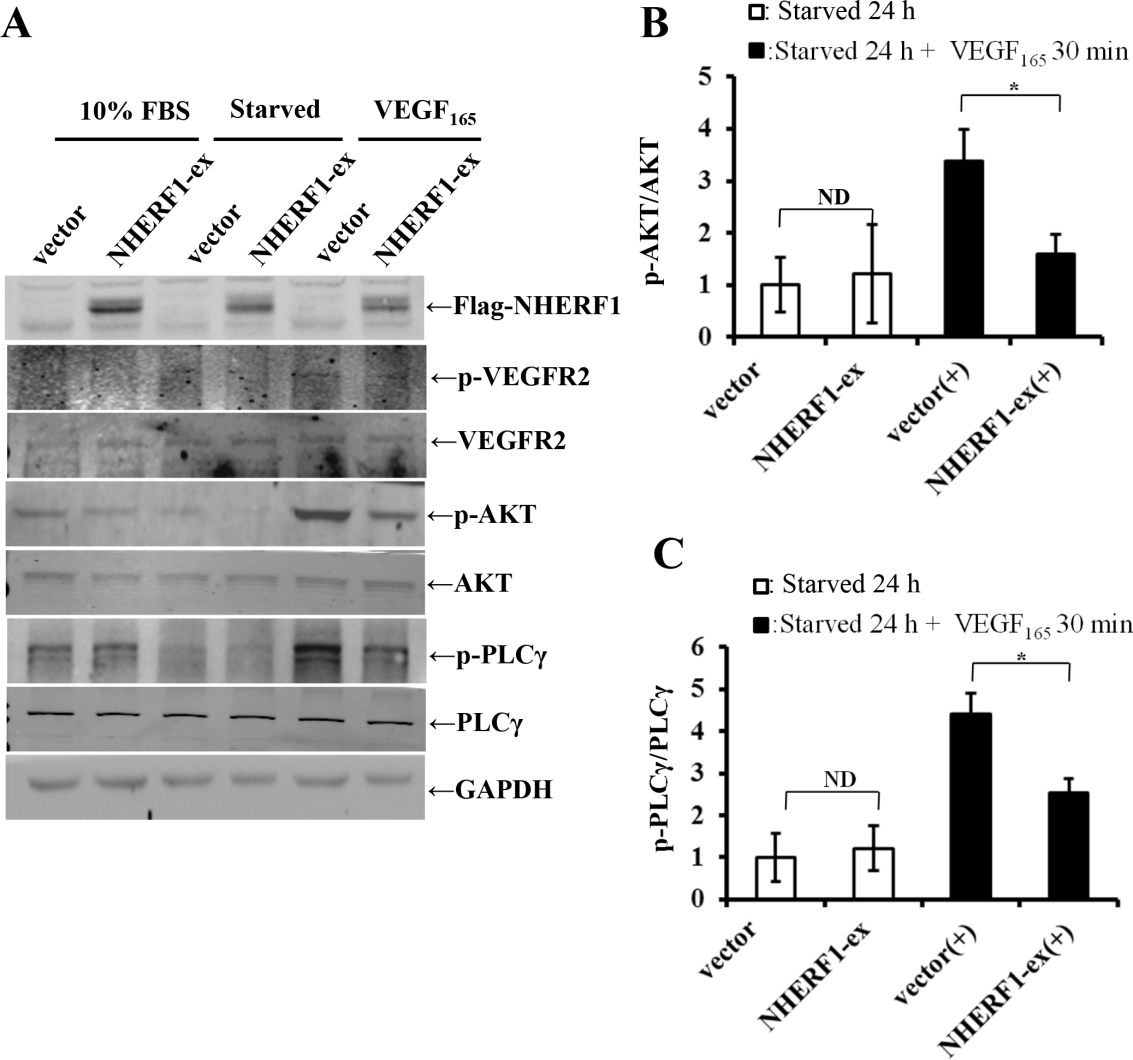
NHERF1 inhibited the phosphorylation activation of VEGFR2 and its downstream AKT and PLCγ activation induced by VEGF165 RKO cells transfected with Flag-NHERF1 plasmid were serum-free starved 24 h, and then stimulated with or without VEGF_165_ 25 ng/ml for 30 min. Immunoblotting was performed using anti-p-VEGFR2, anti-VEGFR2, anti-pS473AKT, anti-AKT, anti-p-PLCγ, and anti-PLCγ antibodies (**A)**. The signals were quantified by densitometry (**B**, **C)**. The results represent the mean values ± SD of three independent experiments. *p < 0.05, **p < 0.01

Since NHERF1 can interact with VEGFR2 in cells, it was reasonable to assume that NHERF1 could also affect VEGFR2 signaling. To test this hypothesis, we detected VEGF_165_-induced phosphorylation activation of VEGFR2, AKT and PLCγ in RKO cells. VEGFR2, AKT and PLCγ were all dramatically phosphorylated in RKO vector control cells following 30 min stimulation with 25 ng/ml VEGF_165_. NHERF1 overexpression significantly blocked VEGF_165_-induced VEGFR2 phosphorylation and inhibited the downstream AKT and PLCγ activation (Figure 5). Meanwhile the base levels of VEGFR2, AKT and PLCγ activation were not detectably different in cells transfected with NHERF1 plasmid or vector control.

### NHERF1 inhibited the migration and invasion of RKO cells through VEGFR2 signaling pathway

**Figure 6:**
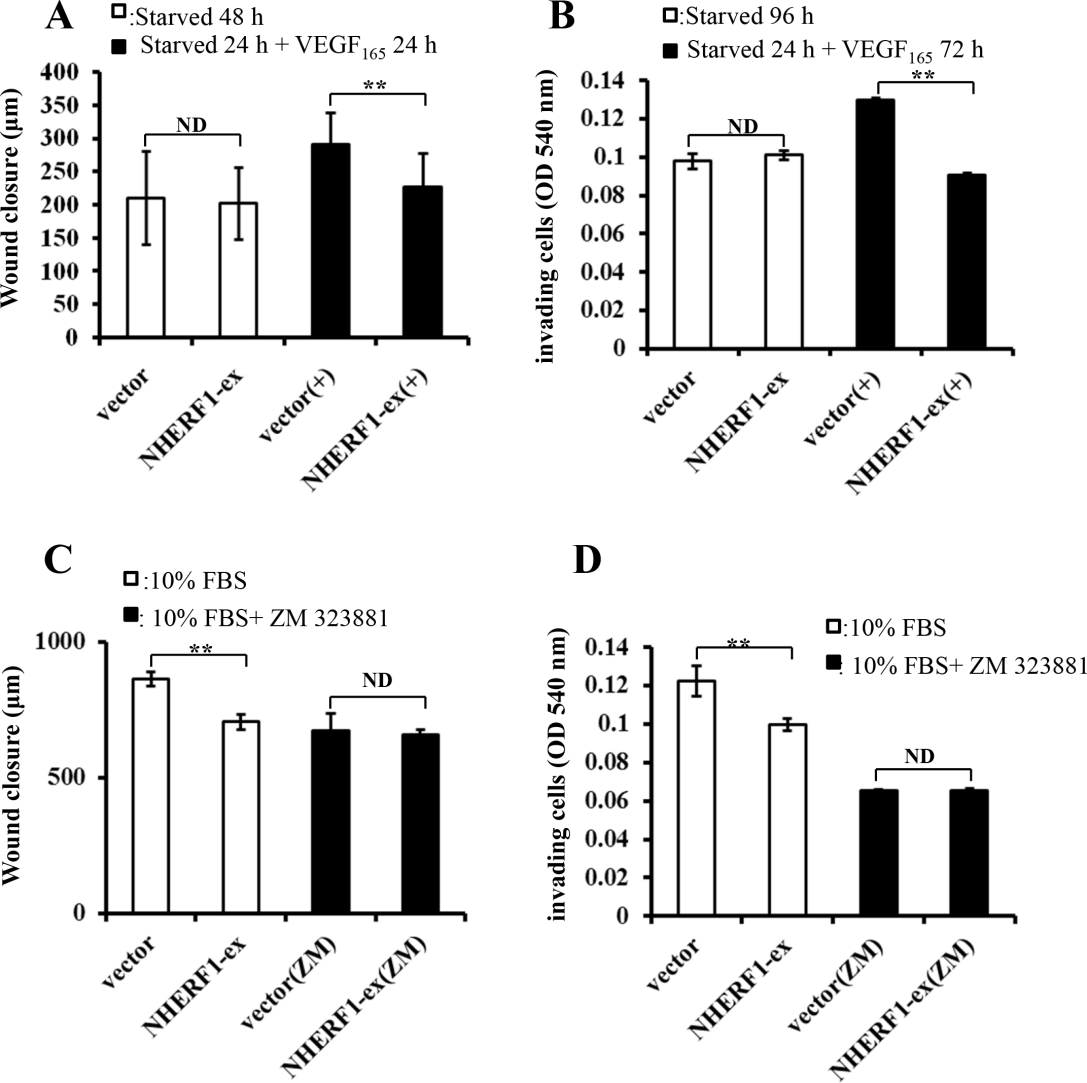
NHERF1 inhibited the migration and invasion of RKO cells through VEGFR2 signaling pathway RKO cells transfected with Flag-NHERF1 plasmid were serum-free starved 24 h, and then cultured with or without VEGF_165_ 25 ng/ml. NHERF1 significantly inhibited cell migration (**A**) and invasion (**B**) induced by VEGF_165_. RKO cells transfected with NHERF1 plasmid were cultured in DMEM supplemented with 10% FBS with or without ZM-323881, VEGFR2 special inhibitor. ZM-323881 (10 nM) removed the inhibitory effects of NHERF1 on cell migration (**C**) and invasion (**D**) The results represent the mean values ± SD of three independent experiments (C, D). *p < 0.05, **p < 0.01

VEGF_165_-induced cell migration and invasion were investigated in RKO cells transfected with NHERF1 plasmid and vector control, in order to explore whether the potential signaling transduction pathway was modulated by NHERF1. After pre-culture in serum-free medium 24 h, cells were treated with 25 ng/ml VEGF_165_. NHERF1 significantly inhibited the migration (Figure 6A) and invasion (Figure 6B) rate of RKO cells induced by VEGF_165_ compared with vector control cells. On the contrary, VEGFR2 special inhibitor ZM-323881 removed the inhibitory effects of NHERF1 on the cell migration and invasion (Figure 6C, 6D). Similar results were also observed in HRT-18 cells (Supplementary Figure S1C, S1D).

## DISCUSSION

The VEGF/VEGFR signaling pathway is involved in cancer-related biological functions and is a therapeutic target in cancer [11]. Anti-VEGF treatment has proved efficacious and improves survival in CRC [12]. However, many patients progress after initial disease stabilization due to acquired resistance to anti-VEGF/VEGFR drugs [14], suggesting that there is complicated regulation of VEGF/VEGFR pathway *in vivo*. Despite rapid progression in understanding the biology and the clinical significant of angiogenesis, there is very little information on the regulation of VEGF/VEGFR pathway specifically to tumor cells.

In the present study, a high level expression of NHERF1 was found in colorectal cancer tissues and probably contributed to the malignant phenotypes of tumor recurrence and distant metastasis (Table 1). More interestingly, it was showed the expression of NHERF1 was positively correlated with VEGFR2 expression (Table 2). High expression of VEGFR2 was associated with shorter overall survival of patients with colorectal cancer depending on the low NHERF1 expression (Figure 1B, 1C). The overall survival rate analysis suggested NHERF1 could play an important role in the progression of colorectal cancer regulated by VEGFR2 signaling pathway. In the course of tumorogenesis, hypoxia becomes an essential feature of the neoplastic microenvironment, typically caused by inadequacy of the local vasculature to supply sufficient oxygen and nutrients to rapidly growing tumor cells [5]. HIF-1α is recognized as an important regulatory protein in the transcription of a variety of tumor related factors including VEGF-A and VEGFR2. VEGF-A in turn promotes the expression of HIF-1α. This is a positive feedback regulation pathway of tumor cells for adaptation to hypoxia microenvironment. Recently, it was also reported that the expression of NHERF1 was strongly correlated with higher expression of HIF-1α in colorectal cancers [31], suggesting association with the tumor hypoxia microenvironment. In the present study, the up-regulation of NHERF1 induced by hypoxia was found in RKO cells by strong correlation with the expression of VEGFR2 and HIF-1α (Figure 2A). The up-regulation of NHERF1 was removed by ZM-323881, VEGFR2 special inhibitor, suggesting that the up-regulation of NHERF1 could be dependent on activation of VEGFR2 signaling pathway (Figure 2B).

VEGF through the high affinity receptor tyrosine kinase VEGFR acts as a high inducer of the oncogenic cascades including Ras/MEK/ERK, PLC-γ-IP3, PI3K/AKT to stimulate cell mitogenesis and cell migration [35]. In addition, the recent study showed that the expression of NHERF1 was increased by nuclear transcription factor NF-κB in the primary macrophages and vascular smooth muscle cells [36]. In colon cancer cells, NHERF1 expression could be increased by the activation of NF-κB signaling and/or the multiple downstream signaling pathway of HIF-1α/VEGF-A induced in the hypoxia microenvironment. This up-regulation of NHERF1 was rapid for hypoxia (within 30 min) (Figure 2A, 2C) suggesting that NHERF1 could serve as a rapid response element to the tumor hypoxia microenvironment and might contribute to the regulation to the VEGF/VEGFR signaling pathway in driving metastatic progression.

Clinical studies show an association between NHERF1 overexpression and malignant progression of colorectal cancers; however, there is very little information on the function of NHERF1 in *in vitro* studies specifically in colorectal cancer cells. In the present study, *in vitro* functional experiments showed that overexpression of NHERF1 reduced cell migration and invasion of RKO cells, and knockdown expression of NHERF1 enhanced the migratory and invasive ability of RKO cells, indicating a tumor suppressor role of NHERF1 in colon cancer cells specifically for metastasis (Figure 3).

In addition to the VEGF receptors, Neuropilin-1 (NP1) has been identified as a co-receptor for VEGF [37]. NP1 enhances the affinity of VEGF_165_ to VEGFR2 and increases its phosphorylation, thereby enhancing downstream signaling [37, 38]. NHERF1, as a PDZ protein, might interact with NP1 through its PDZ-binding domain in the cytoplasm and further form a protein complex with VEGFR2, which could result in the regulation of the downstream signaling. This hypothesis was confirmed by the assays of GST-pull down, immunoprecipitation and immunofluorescence. Results showed NHERF1 could interact with VEGFR2 in cells (Figure 4). The results of western blot (Figure 5) and *in vitro* functional experiments (Figure 6) showed NHERF1 reduced the migratory and invasive ability of RKO cells by inhibition of VEGF signaling pathway. These results suggested a negative feedback in colon cancer cells, in that the activation of VEGF signal pathway increased the expression of NHERF1 and NHERF1 in turn inhibited the phosphorylation activation of VEGFR2 and its downstream signals activation, resulting in reduction of the migration and invasion of colon cancer cells. Anti-VEGF/VEGFR drugs, targeted to suppress the tumor progression through anti-angiogenesis, in fact function both on tumor vascular endothelial cells and cancer cells. The up-regulation of NHERF1 exposed to hypoxia was also inhibited by the anti-VEGF/VEGFR drugs in cancer cells. The negative feedback of NHERF1 to VEGF signaling pathway was disrupted, which could contribute to tumor progression, resulting in acquired resistance to anti-VEGF/VEGFR drugs.

Overall, in the present study, a high level expression of NHERF1 was found in colorectal cancer tissues and it was shown that expression of NHERF1 was positively correlated with VEGFR2 expression. NHERF1 expression induced by exposure to hypoxia could be dependent on the activation of VEGF signaling. NHERF1 in turn inhibited the activation of VEGFR2 signaling pathway which could be due to the interaction between NHERF1 and VEGFR2, resulting in the reduction of the migration and invasion of colon cancer cells. The present results suggest a dynamic interplay between NHERF1 and VEGFR2 signaling in colon cancer cells, which could explain the up-regulation of NHERF1 in colorectal cancer and the contribution of NHERF1 to the regulation of tumor cell responses to the hypoxia microenvironment. The administration of anti-VEGF/VEGFR drugs could cause the downregulation of NHERF1 expression in cancer cells, which could contribute to the progression of tumor and the acquired resistance to drugs. The expression of NHERF1 might be a potential predictive biomarker to identify patients that may benefit from anti-VEGF/VEGFR therapies.

## MATERIALS AND METHODS

### Expression vectors and Small interfering RNAs

The GST-NHERF1 construct was kindly provided by Dr Jiale Dai, MD Anderson Cancer Center, Houston, TX. NHERF1 cDNA was cloned into pReceiver-M12 vector to obtain the 3flag-tagged NHERF1 expression construct, according to the manufacturer's instructions (New England BioLabs, Massachusetts, USA). Small interfering RNA (siRNA) duplexes directed against NHERF1 (siNHERF1-1: 5-GUCGACCACCAGCAGG CGCACGGCGUUG-3; siNHERF1-2: 5-GCUAUGGCUU CAACCUGCATT-3) and the scrambled control RNA(5-UCCAGACGGCGCAGUGGGCGACCGCUAC-3) were synthesized by Invitrogen (California, USA).

### Cell culture and transfection

The human colon cancer cell line, RKO, was obtained from American Type Culture Collection (ATCC, Manassas, VA, USA). Cells were maintained in Dulbecco's modified Eagle medium (DMEM) supplemented with 10% fetal calf serum, penicillin and streptomycin (Gibco BRC, Paisley, Scotland) and were cultured at 37°C in a 5% CO_2_, 95% humidified atmosphere. Cells were transiently transfected with Flag-NHERF1 plasmid and NHERF1-siRNA respectively by using Lipo2000 (Invitrogen).

### Human colorectal specimens

Human colorectal cancer tissues (*n* = 64) and adjacent normal colorectal tissues (*n* = 50) were collected from patients after surgery. These tissues were collected immediately after surgery, and snap-frozen in liquid nitrogen, with the approval of the Local Ethical Committee (This study was conducted between 2002 and 2004 in UHW Cardiff-UK. Ethics were granted by the Cardiff and Vale NHS Trust, project ID: 05/DMD/3562. Full patient consent was obtained). Background normal colorectal tissues (> 2 cm to the tumor) were removed from the same patients. The pathologist verified normal background and cancer specimens, and background samples were confirmed to be free from tumor deposits. The relevant information is provided in Table 1. Survival time was calculated from the date of surgery, and recurrence or metastasis was counted on the date of diagnosis thereof.

### RNA preparation and real-time quantitative polymerase chain reaction (QPCR)

Total cellular RNA was isolated from the homogenized colorectal samples using the ABgene Total RNA Isolation Reagent and following the protocol provided (Advanced Biotechnologies Ltd., Epsom, Surrey, UK). cDNA was generated from 1 ug of each RNA sample and reverse transcribed using a transcription kit (Sigma, St. Louis, MO, USA). Quantitative analysis of NHERF1 mRNA expression in colorectal tissues was determined by QPCR using Amplifor™-based technologies, in which a 6-carboxy-fluorescine-tagged Uniprimer™ (Biosearch Technologies, Inc., Petaluma, CA, USA) was used as a probe together with a pair of target-specific primers and reverse primer with an additional Z-sequence (actgaacctgaccgtaca) (NHERF1 QPCR primers – sense: AGGGAAACTGACGAGTTCTT; antisense: ACTGAACCTGACCGTACATTCACGACTG TTCTCCTTCT). Real-time QPCR conditions were 95°C for 15 min, followed by 60 cycles at 95°C for 20 s, 55°C for 30 s and 72°C for 20 s. The quality of cDNA samples was verified using β-actin as a housekeeping gene (β-actin QPCR primers – sense: CATTAAGGAGAA GCTGTGCT; antisense: ACTGAACCTGACCGTACA GCTCGTAGCTCTTCTCCAG).

### Wound-healing assay

The migratory properties of cells were assessed by wound-healing assay. Cells were seeded at a density of 1.5 × 10^6^ cells/well into a 6-well plate and allowed to reach confluence. The layer of cells was then scraped with a fine gauge needle to create a wound of approximately 1500 μm. Images of the wound were recorded under a phase contrast microscope at different times (0, 6, 12, and 24 h). Wound closure/cell migration was evaluated with motion analysis and line morphometry software (Image J2x).

### Cell invasion assay

Transwell chambers, equipped with a 6.5 mm diameter polycarbonate filter insert (pore size 8 μm) (Becton Dickinson and Company, Oxford, UK), were pre-coated with 50 μg/insert of Matrigel (BD Biosciences). Cells were seeded at a density of 2 × 10^4^ cells/insert. 3 days later, the cells that invade through the Matrigel were fixed, stained, and quantified as described before [39].

### GST pull-down and Western blotting

GST pull-down assays and Western blotting were performed as described previously [40]. The anti-NHERF1 antibody was purchased from BD Biosciences; anti-pS473AKT, anti-AKT, anti-pTyr783PLCγ, anti-PLCγ, HIF-1α and anti-Flag were from Cell Signaling Technology (Danvers, MA, USA); anti-Flk-1 (anti-VEGFR2) and anti-pTyr1214 Flk-1 (anti-pVEGFR2) were from Santa Cruz Biotechnology (Santa Cruz, CA, USA); anti-GAPDH antibody were from ZSGB-Bio (Beijing, China).

### Immunoprecipitation

Cells were harvested and lysed in 1000 ml of ice-cold lysis buffer (10 mM HEPES, 50 mM NaCl, 5 mM EDTA, 1 mM Benzamidine, 0.5% Triton X-100). The lysate was solubilized *via* end-over-end rotation at 4°C for 40 min and clarified *via* centrifugation at 12,000 rpm for 15 min. A small fraction of the supernatant was taken at this point and incubated with SDS-PAGE sample buffer in order to examine expression of proteins in the whole cell extract. The remaining supernatant was divided equally into two tubes, and then incubated with 5 μg antibody and 5 μg IgG (Cell Signaling Technology) respectively with end-over-end rotation at 4°C over night. After incubated with 30 ml of Protein A Sephasose (Ge Healthcare, Chicago, USA) for 3 h with end-over-end rotation at 4°C, the immunoprecipitated proteins were eluted from the beads with sodium dodecyl sulfate (SDS) sample loading buffer, resolved by SDS-PAGE and subjected to Western blot analysis with antibodies.

### Immunofluorescence

Frozen sections of colorectal cancer tissues (*n* = 10) and background normal tissues (*n* = 10) were selected from the tissue bank and cut at a thickness of 6 μm using a cryostat. The sections were fixed with 4% paraformaldehyde in PBS at room temperature for 30min. Sections were incubated for 30 min in 5% BSA blocking solution and probed with monoclonal mouse anti-human NHERF1 primary antibody (1:100) (BD Biosciences) and polyclonal rabbit anti-human VEGFR2 primary antibody (1:100) (Santa-Cruz). This study employed controls that omitted the primary and secondary antibodies. Following extensive washings, sections were incubated with an Alexa Fluor^®^ 488-conjugated anti-mouse IgG (Life Technologies, Massachusetts, USA) at 1:200 dilution and Alexa Fluor^®^ 594-conjugated anti-rabbit IgG (Life Technologies) at 1:200 dilution in the dark for 1 h. After washing three times to remove the unbound secondary antibody, cell nuclei were stained with Hoechst 33258 (Sigma). The sections were finally mounted with FluorSave™ (Calbiochem-Novabiochem Ltd., Nottingham, UK) and visualized with a confocal microscope (Leica Microsystems LAS AF-TCS SP5. Wetzlar, Germany).

### Statistical analysis

SPSS version 16.0 (SPSS, Inc., Chicago, IL, USA) was used for statistical analyses. The results were assessed using non-paired (two-sided) Student's *t*-test and Mann-Whitney *U*-test. The association of expression with the clinical and pathological features was analyzed using one-way ANOVA. The Spearman rank test was used to analyze the correlation between NHERF1 expression and VEGFR2 expression. Overall survival rates were calculated using the Kaplan-Meier method. The log-rank test was utilized to compare the survival rates between groups with varying VEGFR2 expression levels. A *p*-value < 0.05 was defined as statistically significant.

## SUPPLEMENTARY MATERIALS FIGURE


