# Supplementary material for: NHERF1 regulates the progression of colorectal cancer through the interplay with VEGFR2 pathway
Source: Oncotarget. 2016 Dec 15;8(5):7753–65. doi: 10.18632/oncotarget.13949 (PMC5352358; doi:10.18632/oncotarget.13949)
Supplement: Supplementary file 1 [file oncotarget-08-7753-s001.pdf]

NHERF1 regulates the progression of colorectal cancer through the interplay with VEGFR2 pathway

Supplementary Materials

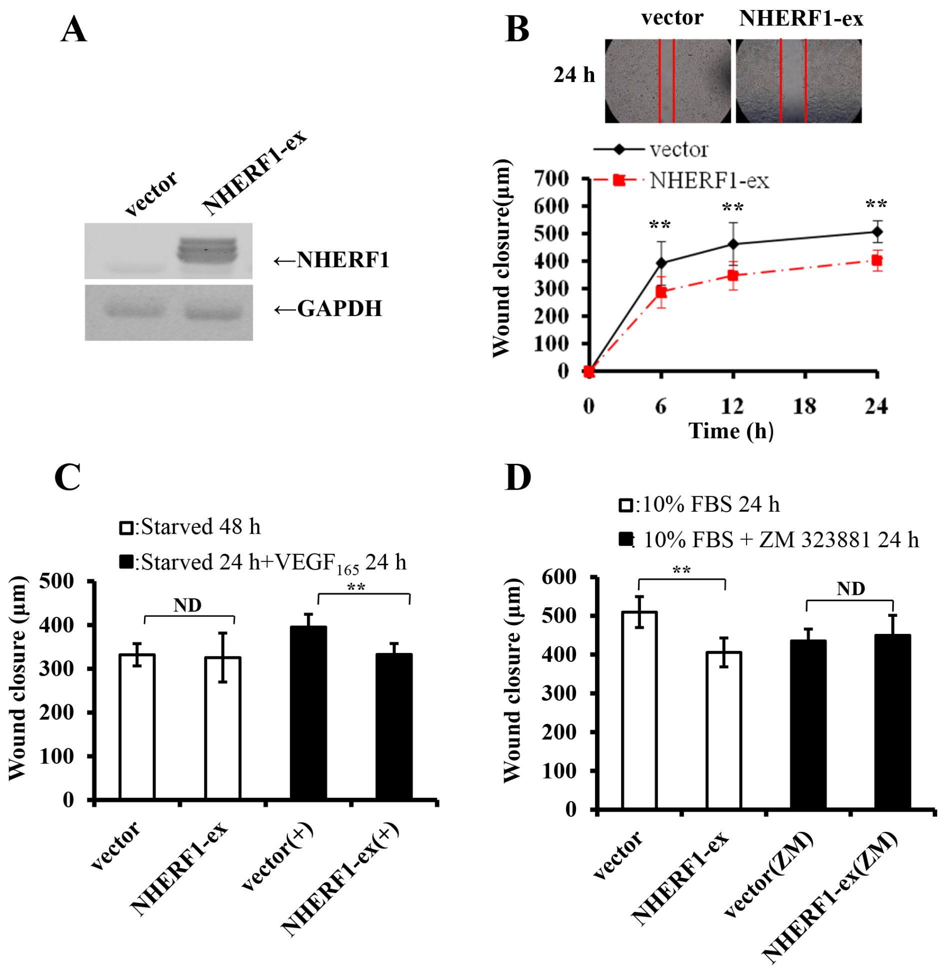

Supplementary Figure S1: NHERF1 overexpression inhibited the migration of HRT-18 cells through the VEGFR2 pathway. NHERF1 plasmid was transfected into HRT-18 cells and the expression of NHERF1 was assessed by Western blotting (**A**) Overexpression of NHERF1 inhibited cell migration (**B**) and $VEGF_{165}$-induced cell migration (**C**) ZM-323881 removed the inhibitory effect of NHERF1 on cell migration (**D**). The results represent the mean values ± SD of three independent experiments (B, C, D). **p* < 0.05, ***p* < 0.01.
